# Albumin mass balance and kinetics in liver transplantation

**DOI:** 10.1186/s13054-018-2053-6

**Published:** 2018-06-07

**Authors:** Mariam Amouzandeh, Greg Nowak, Anna Januszkiewicz, Jan Wernerman, Olav Rooyackers, Åke Norberg

**Affiliations:** 1 Perioperative Medicine and Intensive Care, B31, Karolinska University Hospital, Huddinge, SE-141 86 Stockholm, Sweden; 2 Department of Clinical Science Intervention and Technology (CLINTEC), Karolinska Institutet, Hälsovägen, Stockholm, Sweden; 3 Trauma and Reparative Medicine, Transplantation Karolinska University Hospital, Huddinge, Stockholm, Sweden

**Keywords:** Albumin, Fibrinogen, Capillary leakage, Liver transplantation, Synthesis, Mass balance

## Abstract

**Background:**

In major abdominal surgery albumin is shifted from the circulation, presumably leaking into the interstitial space, contributing to a 30–40% decrease in plasma albumin concentration. During and after liver transplantation exogenous albumin is infused for volume substitution and to maintain plasma albumin concentration. Here we used liver transplantation as a model procedure for the study of albumin mass balance and kinetics during major abdominal surgery with albumin substitution.

**Methods:**

Patients were studied during liver transplantation (*n* = 16), and until postoperative day 3 (POD 3) (*n* = 11). Cumulative perioperative albumin shift was assessed by mass balance of albumin and hemoglobin. Synthesis rates of albumin and fibrinogen were estimated by the flooding technique using deuterium-labeled phenylalanine. Albumin distribution was assessed by radioiodinated human serum albumin.

**Results:**

At the end of surgery, 37 ± 17 g of albumin (*p* < 0.0001) had shifted from plasma, and this amount was stable until POD 3 (48 ± 33 g, *p* = 0.0017 versus baseline). There was 91 ± 37 g exogenous albumin infused peroperatively and another 47 ± 35 g was infused postoperatively until POD 3. Absolute synthesis rates of albumin and fibrinogen on POD 3 were 239 ± 84 mg/kg body weight/day and 33 mg/kg body weight/day (range 5–161), respectively.

**Conclusions:**

Albumin net leakage from plasma progressed until the end of surgery, and was then unaltered until POD 3. This is in contrast with the normalization of the cumulative albumin shift identified at day 3 after non-transplant major abdominal surgery. Liver synthesis of export proteins was high compared to reference values at the third postoperative day, suggesting rapid recovery of synthesis capacity.

**Trial registration:**

Swedish Medical Product Agency, EudraCT 2015-002568-18. Registered on 15 July 2015.

## Background

Intravenous administration of albumin has been used for more than 60 years in different states of circulatory instability. The strong association between body weight gain due to fluid retention and poor outcome following surgery [1] and in critical illness [2, 3] encouraged the use of colloids, such as albumin, with the goal of decreasing the amount of fluids that need to be intravenously administered to obtain circulatory stability. This volume-saving effect has been repeatedly demonstrated in volunteers [4], but is less extensive during surgery, with a ratio between colloids and crystalloids of 1:1.8 [5], and in the critically ill a ratio of 1:1.4 [6]. Although positive short-term effects on circulatory stability are evident [7], long-term effects with a possibility of adverse outcomes are poorly understood and sparsely investigated.

Low plasma albumin concentration (P-alb) is often seen after major abdominal surgery [8, 9]. P-alb below 25 g/L on postoperative day 1 (POD 1) after pancreaticoduodenectomy predicts any serious complication (Clavien grade III–V [10]) [11]. Likewise, a decrease in P-alb ≥ 10 g/L on POD 1 is associated with threefold increased risk of any postoperative complication [8]. The time course of the perioperative decrease in P-alb is well-characterized and occurs mainly during the first hours of surgery [12, 13]. The capillary leakage in the perioperative setting can be studied by the transcapillary escape rate (TER) of radioiodinated human serum albumin [14]. The mechanism of impaired capillary patency is unclear, but inflammation and fluid overload are possible contributors [15]. By keeping rigorous track of infusions of exogenous albumin and losses of albumin by bleeding and in drains, we have adapted a method of mass balance. Using this approach, we demonstrated a cumulative perioperative albumin shift from plasma of 24 ± 17 g, presumably to the interstitial space, 1 h after the end of major abdominal surgery [13]. However, whether this loss can be affected by intravenous administration of exogenous albumin is not known.

Liver transplantation is a life-saving procedure in end-stage liver disease. In our institution, fluid treatment during this surgical procedure has decreased over time and is now standardized to 5 ml/kg body weight/hour, although extra fluids can be administered at the discretion of the attending anesthesiologist as guided by circulatory monitoring. Concomitantly, bleeding and exogenous blood transfusions have decreased. Still, exogenous albumin is given during and after liver transplantation for volume substitution and with the intention of maintaining P-alb above 25 g/L. This level is suggested to facilitate the interpretation of immune-suppressive drug concentrations. The fate of albumin beyond the initial volume effect is, however, poorly understood. The graft recovery after liver transplantation is monitored by blood chemical analysis, but the recovery of liver function in terms of the synthesis rates of albumin and fibrinogen, two major plasma proteins synthesized in and exported from the liver, has not been investigated previously.

The primary aim of the present study was to investigate the cumulative perioperative albumin shift at the end of surgery for liver transplantation, and on the POD 3, respectively, when a generous amount of exogenous albumin is supplied. The secondary aim was to quantify the synthesis rates of two liver export proteins, albumin and fibrinogen, on POD 3 after liver transplantation.

## Methods

This prospective exploratory study of patients during and after liver transplantation was performed at the Karolinska University Hospital Huddinge between October 2015 and October 2016 in accordance with the Declaration of Helsinki and in compliance with Good Clinical Practice. The study protocol was approved by the Regional Ethical Board in Stockholm (2015/1048-31/2) and by the Swedish Medical Product Agency (EudraCT 2015-002568-18). The study was monitored by the Karolinska Trial Alliance. All patients gave written informed consent after being informed orally and in writing about the investigational procedure and the possible risks involved.

### Patients

**Fig. 1:**
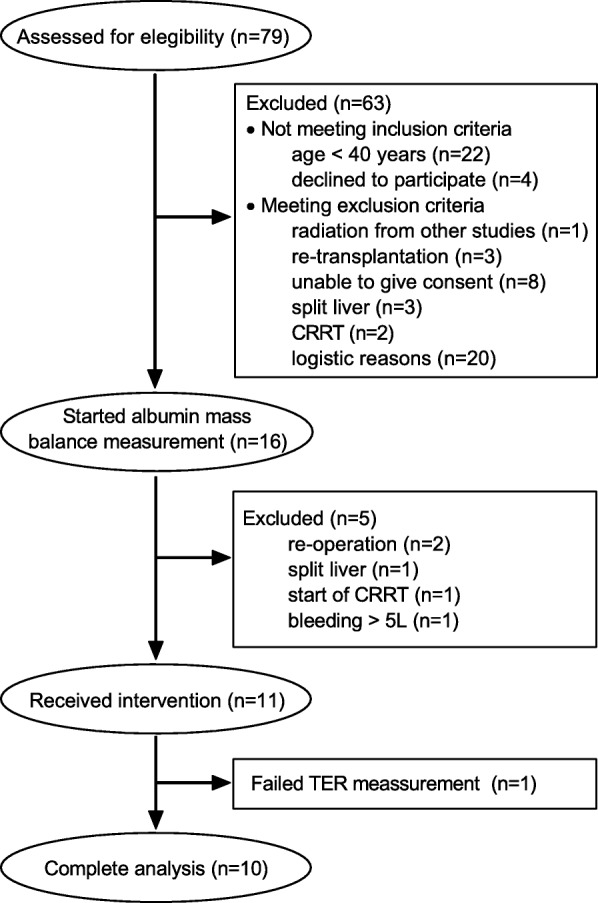
Consolidated Standards of Reporting Trials (CONSORT) diagram of patient recruitment. CRRT, continuous renal replacement therapy; TER, transcapillary escape rate

During the study period, liver transplantation was performed on 79 occasions, and 16 subjects (6 female) aged 53 ± 7 years, body weight 82 ± 24 kg, body mass index 26.8 ± 7.0 kg/m^2^, were recruited to the study (Fig. 1). All subjects were evaluable until the first primary end point at the end of surgery (EOS), and finally 11 subjects received the isotopic intervention on POD 3. In one patient the TER measurement failed to reach the pre-set acceptable quality (a regression coefficient exceeding 0.85), but this patient was included in all other analyses. Patients younger than 40 years and pregnant women were excluded from the study based on the recommendation by the World Health Organization (WHO), that the risk of potassium iodine treatment after nuclear accidents exceeds the benefit if the radiation dose is low in patients ages 40 and higher [16]. Pregnancy tests were performed in women < 55 years old. Patients who had participated in other studies involving radiation or the use of stable isotopes within 30 days were excluded, as were patients undergoing re-transplantation, graft size reduction, bleeding more than 5000 ml, or continuous renal replacement therapy.

The patients had a model for end-stage liver disease (MELD) score of 14 ± 5 [17], and according to the Child-Pugh classification for cirrhosis mortality there were three patients in group A, nine in group B, and four in group C [18]. The main indication for liver transplantation was hepatocellular carcinoma in six patients, primary biliary cirrhosis in three patients, alcoholic cirrhosis in three patients, Morbus Osler disease in one patient, autoimmune hepatitis in one patient, non-alcoholic steatohepatitis in one patient, and cryptogen cirrhosis in one patient. The following comorbidities were noted: ulcerative colitis (*n* = 3), Crohn’s disease (*n* = 2), hepatitis C (*n* = 2), diabetes mellitus (*n* = 2), obesity (*n* = 2), renal insufficiency (*n* = 2), chronic obstructive pulmonary disease (*n* = 2), asthma (*n* = 1), peribronchial fibrosis (*n* = 1), hypertension (*n* = 1), and hypogammaglobulinemia (*n* = 1).

### Study procedure

All liver transplantations were performed using liver grafts from brain-dead donors. All patients had an arterial line and central venous catheters, and circulation was further monitored by pulmonary artery catheter (Vigilance II monitor, Edwards Lifesciences, Irvine, CA, USA) and/or trans-esophageal echocardiography for guidance of fluid therapy and use of vasopressor. An autologous blood recovery system (Cell Saver® 5+, Haemonetics Corp, Braintree, MA, USA) was used in all patients without hepatocellular carcinoma. General anesthesia was maintained by sevoflurane in oxygenated air at a minimal alveolar concentration of 0.8–1.0, complemented with fentanyl. Intravenous fluids were administered according to the unit routines i.e. albumin 50 mg/ml at 2 ml/kg body weight/h (Baxter Medical AB, Kista, Sweden), acetated Ringer’s solution 2 ml/kg body weight/h and buffered glucose 25 mg/ml at 1 ml/kg body weight/h. Extra fluids were administered at the discretion of the attending anesthesiologist. Most patients had a norepinephrine infusion to keep mean arterial blood pressure above 60 mmHg.

Blood sampling for mass balance analysis commenced in the operating room as soon as there was an arterial line, and blood was then sampled at approximately hourly intervals during surgery i.e. at the same time intervals as routine blood gases. Samples were also taken from all administered blood products including salvaged blood, and from heparinized suction bottles, for determination of albumin and hemoglobin. After the end of surgery similar samples were taken from blood, from drains and from blood products given intravenously, two to three times per day until POD 3. The amounts of fluid were weighed. Samples were taken in tubes with sodium heparin, spun at room temperature at 2000 g, and plasma was then stored at − 18 °C until nephelometry analysis at Studiecenter Karolinska University Hospital. The details and applied equations for the mass balance calculations have been published before [13]. Briefly, mass balance analysis compares the dilution of hemoglobin with that of albumin, whilst keeping track of all measured gains and losses of the two compounds. If albumin is diluted more than hemoglobin, this suggests that albumin has been shifted from plasma, presumably to the interstitial space. The measured plasma volume at POD 3 was used for adjustment of baseline plasma volume in the 11 patients that had such a measurement, otherwise anthropometric plasma volume was calculated and used as the baseline in the calculations [19]. Ascites was measured separately in the assessment of perioperative fluid balance and body weight changes, and was not included in the mass balance analysis. Differences in the weight of native and donor livers were also analyzed in changes of body weight. Perspiration was assumed to be 3 ml/kg body weight/h or 0.5 ml/kg body weight/h during surgery and postoperatively, respectively. Differences between estimated bleeding during surgery and the weighing of sponges and suction bottles were treated as perspiration in the fluid balance calculations.

On POD 3 albumin and fibrinogen synthesis rates were measured in the semi-recumbent position. Nutrition was limited to glucose 50 mg/ml at 80 ml/h during 4 h prior to the investigation. Synthesis rates were measured by the flooding dose technique, previously explained in detail [12, 20, 21], using a 10 min infusion of L-^2^H_5_ phenylalanine 20 mg/ml, 10 mol percent excess, at a dose of 0.45 g/kg. Thereafter blood was sampled at 0, 5, 15, 30, 50, 60, 70, and 90 min after the start of the infusion for determination of the mol percent excess of L-^2^H_5_ phenylalanine to phenylalanine as an index of the precursor pool. After separation of albumin from the other plasma proteins, the synthesis rate was calculated from the rate of incorporation of L-^2^H_5_ phenylalanine into albumin in the samples taken at 50, 60, 70, and 90 min. Analysis was performed on a quadrupole gas chromatography mass spectrometer (Agilent, Kista, Sweden). The fractional synthesis rate (FSR), i.e. the fraction of the intravascular pool that is synthesized every day, was calculated as previously described [12], and the absolute synthesis rate (ASR) was calculated as the product of FSR and the total plasma pool of albumin obtained from plasma volume and P-alb. The principle of determination of fibrinogen FSR and ASR was similar to that of albumin, although blood samples were taken in citrate tubes, but the process of separation of fibrinogen from other plasma proteins was different from that of albumin [22].

Measurement of TER and plasma volume was performed using radiolabeled albumin (SERALB-125, CIS bio international, Gif-sur-Yvette Cedex, France). A dose of 200 kBq was injected intravenously 20 min after the start of the stable isotope procedure described above, followed by blood sampling on nine occasions during 70 min. The radioactivity was measured by scintillation counting (Wallac Compu-gamma CS 1282, energy window 20-82 keV) at the Department of Nuclear Medicine at Karolinska University Hospital Huddinge. TER was derived from the slope of the logarithm of counts per minute versus time plot. Plasma volume was calculated from the radioactive dose administered divided by the calculated activity in plasma at time of the injection, obtained from back extrapolation of the slope used for the TER assessment [9].

### Statistics

Data are presented as mean ± standard deviation or median (range) as appropriate according to the Shapiro-Wilk normality test. When comparing two time points, the dependent *t* test was used. Repeated measures analysis of variance (ANOVA) of the three main time points (baseline, EOS, and POD 3) was performed (*n* = 11) using the Geissner-Greenhouse correction i.e. not assuming sphericity, and Tukey’s post-hoc multiple comparisons test comparing every mean to every other mean for parameters where normality could not be excluded according to the Shapiro-Wilk test. Otherwise, Friedman’s non-parametric ANOVA with Dunn’s post-hoc test was used. Correlation is presented as Pearson’s coefficient of correlation or Spearman’s rank correlation coefficient as appropriate. The 95% confidence intervals of the means are given for the most important outcome parameters.

A study population of 10 was chosen to achieve 80% power to detect a within-subject albumin shift from plasma at the end of surgery with an effect size of 1.0 using the two-sided *t* test and a significance level of 5%. In the absence of published data on patients undergoing liver transplantation, we looked at our previous study in patients undergoing major abdominal surgery where the albumin shift was 24 ± 17 g, corresponding to an effect size of 1.4 [13]. GraphPad Prism 7.02 (GraphPad Software, La Jolla, CA, USA) was used for the statistical analysis and PASS 2008 (NCSS LLC, Kaysville, UT, USA) was used for power analysis.

## Results

### Albumin mass balance

**Fig. 2:**
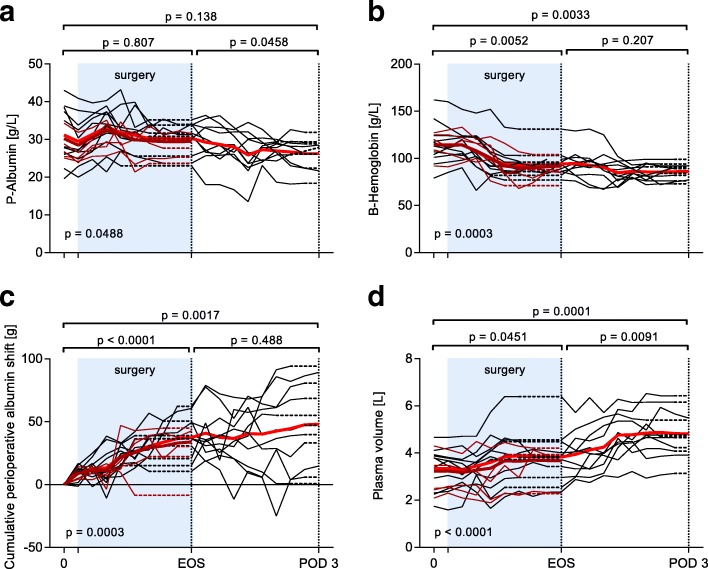
Temporal pattern of plasma albumin (**a**), blood hemoglobin (**b**), cumulative perioperative albumin shift (**c**), and plasma volume (**d**) during and after liver transplantation. Bold red line denotes mean in 11 patients; bold dark red line denotes mean in 16 patients: the 5 patients excluded after surgery are marked by thin dark red lines. Dotted lines are used to carry values forward to the primary endpoints. The *p* values are for repeated measures analysis of variance with Tukey’s post-hoc adjustment for multiple comparisons in 11 patients. EOS, end of surgery; POD 3, postoperative day 3

P-alb (*n* = 11) was 31.2 ± 7.7 g/L before surgery, 30.3 ± 4.3 at EOS, and 26.2 ± 4.0 at POD 3 (Fig. 2a); this corresponded to a total decrease of 12 ± 21%, which was not statistically significant. During surgery the total exogenous albumin administration was 91 ± 37 g including that in salvaged blood and other blood products, and the loss of albumin in bleeding amounted to 42 ± 36 g. Postoperatively exogenous albumin administration was 47 ± 35 g, and the measured loss in drains was 25 ± 23 g between EOS and POD 3 (Table 1). Blood hemoglobin was 113 (79; 162) before surgery and 89 (73; 99) at POD 3, corresponding to a decrease by 22 ± 16% (Fig. 2b).

**Table 1:** Albumin and fluid balance during two perioperative time periods in patients undergoing liver transplantation

	Day of surgery until EOS, *n* = 11	EOS until POD 3, *n* = 11	Day of surgery until EOS, *n* = 11 + 5
Albumin (g)			
Albumin loss in bleeding and drains	−42 ± 36	−14 (− 84; − 6)	− 43 ± 35
Albumin administered	91 ± 37	47 ± 35	87 ± 38
Albumin balance	49 ± 18	22 ± 26	45 ± 18
Albumin shift	37 ± 17	11 ± 30	34 ± 19
Fluids (L)			
Blood and drain losses	1.87 ± 1.46	0.68 (0.10; 4.68)	1.98 ± 1.58
Urine	0.40 ± 0.24	6.39 ± 3.56	0.34 ± 0.22
Perspiration^a^	2.02 ± 0.68	1.97 ± 0.32	2.08 ± 0.73
Sum of fluid losses	4.30 ± 1.94	9.59 ± 3.84	4.40 ± 2.15
Albumin and blood products	1.96 (0.76; 4.80)	0.45 (0.15; 2.47)	1.83 (0.75; 4.88)
Crystalloids and glucose	3.98 ± 1.16	7.24 ± 2.17	3.87 ± 1.10
Dextrane	0.00 (0; 0.44)	0.93 (0.06; 1.47)	0.00 (0; 0.44)
Sum of infusions	6.20 ± 2.09	11.28 ± 3.18	6.09 ± 2.16
Fluid balance	1.90 ± 1.04	1.54 ± 4.58	1.69 ± 1.14

Values are presented as median (range) or mean ± standard deviation, as appropriate

*EOS* end of surgery, *POD 3* postoperative day 3 at end of isotope measurement

^a^During surgery perspiration is the sum of 3 ml/kg body weight/hour and weight of fluid loss in sponges and suction bottles not explained by bleeding

The cumulative perioperative albumin shift, the primary end point of the study, was 37 ± 17 g at EOS (*p* < 0.0001, ANOVA; 95% confidence interval of the mean net leakage 23–58 g), and 48 ± 33 g on POD 3 (95% confidence interval versus baseline 21–75 g, *p* = 0.0017 and versus EOS − 14 to 35 g, *p* = 0.49). However, the time course differed between the individual patients (Fig. 2c). Plasma volume increased over time, also in the postoperative period (Fig. 2d). There was no statistically significant correlation between the cumulative perioperative albumin shift at EOS and peroperative albumin administration (*p* = 0.098, *n* = 11). Data for the whole group of 16 patients that had complete data until the EOS were overall very similar to the subgroup of 11 patients (Fig. 2a-d and Table 1), with the primary outcome cumulative correlation between the cumulative perioperative albumin shift and peroperative albumin administration at the EOS in 16 patients (*r*^2^ = 0.34, *p* = 0.018).

### Surgery and fluid balance

The duration of surgery was 5.5 ± 1.5 h and the length of the procedure was statistically correlated with the cumulative perioperative albumin shift (*p* = 0.019), albumin loss (*p* = 0.031), and the amount of albumin administered (*p* = 0.013). Surgical bleeding amounted to 1.87 ± 1.46 L. The donor liver had a weight of 1.89 ± 0.62 kg, similar to the 1.87 ± 0.64 kg of the recipient’s original liver, with a difference of 0.02 ± 0.63 kg. Liver donors were aged 64 ± 14 years, had body mass index of 26.6 ± 4.2 kg/m^2^, cold ischemic time was 7.8 ± 1.4 h, and liver perfusion after vessel implantation was 0.28 ± 0.14 L/min in the hepatic artery and 1.78 ± 0.76 L/min in the portal vein. Fluid balance was positive at the EOS (Table 1), but variable after that time point. The cumulative changes in fluid balance and body weight are presented in Table 2. Data on blood chemical analysis from the hospital laboratory are presented in Table 3. Four patients had between 2 L and 3 L of ascites removed at the start of laparotomy. Among the remaining patients, only three had “no ascites”. When comparing estimated bleeding to the weighing of suction bottles and sponges, there was a discrepancy of 0.71 ± 0.57 kg, classified as perspiration in the fluid balance calculation.

**Table 2:** Cumulative balance of albumin, fluid balance, and changes in body weight in patients (*n* = 11) undergoing liver transplantation

	End of surgery	Postoperative day 3	ANOVA
Albumin balance (g)	49 ± 18 *p* < 0.0001^a^	71 ± 33 *p* = 0.041^a^, *p* < 0.0001^b^	*p* < 0.0001
Fluid balance (L)	1.90 ± 1.04 *p* = 0.0003^a^	3.44 ± 4.30 *p* = 0.53^a^, *p* = 0.058^b^	*p* = 0.0484
Body weight (kg)	2.39 ± 1.98 *p* = 0.0064^a^	4.73 ± 4.05 *p* = 0.15^a^, *p* = 0.0080^b^	*p* = 0.0033

Values are presented as mean ± standard deviation or median (range). The baseline is zero in all parameters but is not presented. Baseline body weight is adjusted for differences in weight of the recipient’s original liver and the donor liver, and for the major amounts of ascites

*ANOVA* analysis of variance

^a^The *p* value for comparison with the previous time point

^b^The *p* value compared to baseline. Statistical analysis was performed by repeated measures ANOVA with Tukey’s test for post-hoc multiple comparisons between all three measurement time points

**Table 3:** Laboratory tests in patients (*n* = 11) undergoing liver transplantation

	Before Surgery	End of surgery^a^	POD 3
Blood hemoglobin (g/L)	113 (86; 166)	97 (77; 128)*	90 (72; 98)
Blood hematocrit (fraction)	0.339 ± 0.060	0.290 ± 0.043**	0.253 ± 0.032*
Blood thrombocyte count (*10^9^/L)	93 (57; 313)	104 (55; 187)	56 (32; 184)**
Blood white blood cell count (×10^9^/L)	5.7 ± 2.2	9.1 ± 3.4**	9.8 ± 4.2
Plasma C-reactive protein (mg/L)	4 (0.9; 39)	47 (19; 118)***	23 (11; 67)
Plasma creatinine (μmol/L)	75 (51; 126)	80 (49; 117)	83 (41; 373)
Plasma bilirubin (μmol/L)	25 (4; 195)	54 (28; 130)	25 (9; 131)*
Plasma aspartate transaminase (μkat/L)	1.14 (0.54; 3.19)	30 (6.1; 95)***	7.46 (2.88; 51)
Plasma alanine aminotransferase (μkat/L)	0.70 (0.34; 2.67)	18 (4.2; 61)***	9.47 (1.82; 44)
Prothrombine time - international standardized ratio	1.4 (1.0; 1.8)	2.3 (1.3; 3.3)**	1.5 (1.1; 3.6)**
Blood glucose (mmol/L)	5.7 (4.7; 19.4)	12.2 (10.8; 16.4)**	7.8 (4.9; 14.8)

*POD 3* postoperative day 3

^a^End of surgery samples are taken at the intensive care unit at arrival, approximately 1 h after end of surgery, except for plasma C-reactive protein taken at 6 a.m. the next day. Statistical analysis was performed by repeated measures analysis of variance with Tukey’s post-hoc multiple comparisons test or Friedman’s test with Dunn’s multiple comparisons test as appropriate

*p < 0.05, **p < 0.01, and ***p < 0.001 compared to the preceding measurement time point

### Albumin synthesis and distribution

**Fig. 3:**
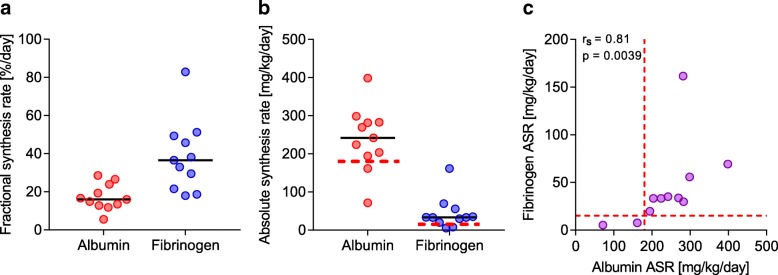
Liver-export protein synthesis after liver transplantation on postoperative day 3. **a** Fractional synthesis rates of albumin (red) and fibrinogen (blue); median value is marked as a line. **b** Absolute synthesis rates of albumin (red) and fibrinogen (blue). **c** Spearman rank correlation between absolute synthesis rates (ASR) of albumin and fibrinogen, respectively. Red dotted lines denote normal values in healthy volunteers (**b** and **c**) [22]

The synthesis measurements on POD 3 started at 62 ± 7 h after the EOS, most often early in the morning due to logistic feasibility. Albumin kinetic parameters are summarized in Table 4. The albumin fractional synthesis rate was 17.2 ± 6.9% per day and the corresponding albumin absolute synthesis rate was 239 ± 34 mg/kg body weight/day. The fibrinogen fractional synthesis rate on POD 3 was 36.5% (17.9–82.9) per day and the corresponding absolute synthesis rate was 33 (5; 161) mg/kg body weight/day (Fig. 3a b and). Albumin and fibrinogen absolute synthesis rates were positively correlated (Fig. 3c).

**Table 4:** Albumin kinetic parameters on postoperative day 3 after liver transplantation

Variable	Value
Plasma albumin concentration (g/L)	26.4 ± 4.1
Plasma volume (L)	4.81 ± 1.02
Blood volume (L)	6.28 ± 1.28
Intravascular albumin mass (g)	128 ± 39
Cumulative perioperative albumin shift (g)	48 ± 33
Fractional synthesis rate (%/day)	17.2 ± 6.9
Absolute synthesis rate (mg/kg/day)	239 ± 84
Secretion time (min)	29 (25; 49)
Transcapillary escape rate (%/h)	5.9 ± 2.2
Absolute escape flow rate (g/h)	6.9 ± 2.7

Secretion time is the time after start of tracer infusion when albumin labeled with L-^2^H_5_ phenylalanine starts to appear in peripheral blood

Plasma volume estimated by dilution of ^125^I-HSA was 4.81 ± 1.02 l (*n* = 11) corresponding to 55 ± 13 mL/kg body weight. This is 14% more than by anthropometry according to Nadler [19] (4.22 ± 0.73 L). The mean standard deviation of plasma volume (*n* = 11) by back extrapolation of a single measurement was 27 ± 9 ml, corresponding to a coefficient of variation of less than 1% and a mean 95% confidence interval of 109 ± 35 ml. TER was 5.6 ± 2.0%/h (*n* = 10) with a coefficient of variation of 13%. Intravascular albumin mass, derived by multiplying P-alb by plasma volume, was 128 ± 39 g, whereas the albumin absolute escape rate from plasma was 6.9 ± 2.7 g/h.

**Fig. 4:**
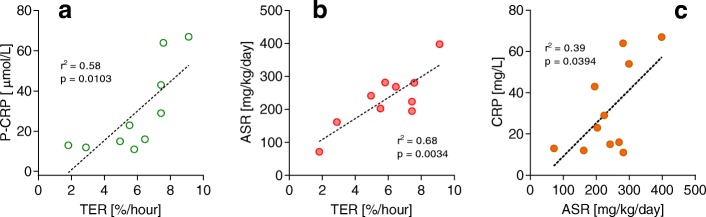
Correlation between plasma C-reactive protein (CRP) and two albumin kinetic parameters in patients 3 days after liver transplantation. **a** CRP versus transcapillary escape rate (TER), **b** Albumin absolute synthesis rate (ASR) versus TER, and **c** CRP versus ASR

In an exploratory analysis TER correlated with both albumin ASR (*p* = 0.003, *r*^2^ = 0.68) and C-reactive protein (*p* = 0.010, *r*^2^ = 0.58) but not with white blood cell count (WBC) count or the cumulative perioperative albumin shift on POD 3. The albumin ASR correlated with plasma C-reactive protein (*p* = 0.039, *r*^2^ = 0.39) but not with WBC on POD 3 (Fig. 4). Also the fibrinogen ASR correlated with C-reactive protein (*p* = 0.016, Spearman’s rank correlation coefficient = 0.72).

In a post-hoc analysis the albumin ASR and fibrinogen ASR were correlated with other laboratory indices of liver function such as plasma prothrombin time, bilirubin, and liver enzymes. The strongest Spearman rank correlation was between the fibrinogen ASR and prothrombin time (*r*_S_ = − 0.424, *p* = 0.192), whereas the *p* value was ≥ 0.5 in all other tests for correlation.

## Discussion

We have taken advantage of the local routine for patients that undergo liver transplantation to study the fate of infused exogenous albumin in conjunction with major abdominal surgery. Albumin was shifted from blood, presumably to the interstitial space, at 37 ± 17 g at the end of surgery. By postoperative day 3 a total of 48 ± 33 g albumin was lost from plasma. The institutional routine of substantial intravenous albumin administration during and after liver transplantation until POD 3 reached a total of 138 ± 55 g, which is of the same magnitude as the measured total intravascular albumin mass. Although the cumulative perioperative albumin shift was somewhat larger than the 24 ± 17 g reported after the end of surgical procedures without albumin supply [13], it is not clear if, or to what degree, the exogenous albumin contributed to this difference in albumin shift. Fluid overload has been suggested to cause capillary leakage, and measured plasma volume at POD 3 was high. Thus, a generous albumin supply might contribute to the loss of albumin from the circulation by its plasma-expanding property. In contrast, albumin has also been suggested to stabilize the endothelial glycocalyx [15], and to block heparin binding protein, which is suggested to be an important factor in endothelial patency [23]. There was no significant correlation between the given amount of exogenous albumin and the cumulative perioperative albumin shift in 11 patients but there was significant correlation in 16 patients. However, *r*^2^ = 0.34 in the test for correlation suggests the presence of other more important mechanisms. Furthermore, the length of surgery correlated with both the amount of exogenous albumin and the cumulative perioperative albumin shift, and is possibly related to inflammation that is another proposed mechanism of capillary leakage [14, 15]. During surgery, the magnitude of systemic inflammation is increased by manipulation of the small intestine [24] and by ischemia-reperfusion injury in the donor liver [25], which might both be associated with surgery time.

The body weight gain of 2.4 and 4.7 kg at the EOS and POD 3, respectively, suggests an increased extravascular space containing albumin at a concentration of 10–15 g/L, which is similar to the interstitial albumin concentration in volunteers [26]. The prevailing increase in albumin shifted from the circulation on POD 3 differs from what is seen in patients undergoing major abdominal surgery without albumin infusion, where the cumulative perioperative albumin shift decreased on POD 3 [13]. Although we aimed at a homogenous population undergoing standard liver transplantation without complications, the variability was considerable among these patients selected for the study. Two subjects had a body mass index under 18 kg/m^2^ and six had body mass index higher than 30 kg/m^2^, bleeding varied from 100 to 5000 ml, and surgery time from 3.1 to 8 h. However, it is also worth noting that the five patients excluded from the postoperative part of the study protocol due to predefined exclusion criteria, did not differ at all at the EOS compared to the 11 patients with full data on the cumulative perioperative albumin shift, P-alb, blood hemoglobin (B-Hb), or plasma volume. In the future, it will be of interest also to explore the outliers after liver transplantation, because postoperative albumin administration is likely to be much higher in some of these patients.

The use of continuous exogenous albumin infusion during liver transplantation is part of a restrictive fluid volume protocol at our institution. Still, fluid shifts are large and crystalloid supply generous enough to cause a significantly positive fluid balance and a weight gain at POD3. Plasma is only given in connection with major bleeding [27], because prophylactic administration of plasma can disturb the rebalance of hemostasis associated with liver failure [28], and all blood products are associated with patient morbidity and impaired graft survival [29]. Compared to our previous study in major abdominal surgery without routine exogenous albumin supply [13], P-alb decreased less during the surgical procedure in our study cohort, but we still lack evidence of any long-term beneficial effects of maintaining P-alb. The high plasma volume compensated for the low P-alb, resulting in a rather normal intravascular albumin mass on POD 3. P-alb thus remains as a weak index of the albumin turnover situation, and a low value can either be the result of increased losses, decreased synthesis, or an increased volume of distribution, both within and outside the vascular space. We speculate that the transient increase in plasma volume in Fig. 2d between POD 1 and POD 2 is associated with occult bleeding. However, this does not affect the cumulative perioperative albumin shift more than marginally, as simulated in the supplement to our previous publication [13].

On POD 3 albumin de novo synthesis was high in both fractional and absolute rates in the liver-transplanted patients. Healthy volunteers have albumin FSR between 7 and 14%/day and ASR between 142 and 239 mg/kg/d [30], and using the same flooding dose technique our research group identified albumin FSR of 14 ± 3%/day and ASR of 177 ± 34 mg/kg body weight/day after a light standardized meal [22]. Also albumin ASR of 184 ± 72 is reported in critically ill patients with multi-organ failure [31]. The strong correlation between the synthesis rates of albumin and fibrinogen in the individual subjects also suggests this to be a general feature of liver function, not influenced by the large exogenous supply of albumin or the absence of exogenous fibrinogen. It also demonstrates quick recovery of liver-export protein synthesis. The rapid growth of liver tissue after liver resection [32] parallels the quick recovery of synthesis capacity in the insulted donor liver, an interpretation that is supported by our findings. In this limited number of patients, albumin ASR and fibrinogen ASR did not correlate with laboratory indices of liver function. Thus, we speculate that capacity for synthesis of liver proteins might serve as a new independent index of liver function after orthotopic liver transplantation.

Alterations in the rates of synthesis and degradation of albumin are not considered in the albumin mass balance calculations. If the synthesis rate during the first few days after surgery is less than the degradation rate, this would cause underestimation of the cumulative perioperative albumin shift by POD 3. In our patients albumin ASR was 20 ± 6 g/day on POD 3, which is high compared to the albumin ASR of 11 ± 4 g on POD 2 after major abdominal surgery [9]. Here the albumin ASR before POD 3 in the liver-transplanted patients and the albumin degradation rate was unknown. However, strong correlation between albumin synthesis and degradation rates is proposed in liver insufficiency [30]. In conclusion, our finding of persistent high cumulative perioperative albumin shift after liver transplantation with generous albumin administration is robust.

The statistical correlation between inflammation reflected by C-reactive protein and TER, previously demonstrated in patients undergoing major abdominal surgery [9], was confirmed in this study (Fig. 4a), and is in line with previous reports on groups of patients with endothelial dysfunction [33]. We speculate that the concomitant correlation between C-reactive protein and the synthesis rates of albumin (Fig. 4c) and fibrinogen, respectively, might partly relate to the fact that C-reactive protein is also mainly synthesized in the liver, and that the value is not only a marker of inflammation but also in part of liver synthesis capacity.

The strength of this study lies in the application of feasible methodology to assess the distribution of exogenous albumin during liver transplantation, a major abdominal procedure. It is also the first assessment of the recovery of export protein synthesis capacity in the transplanted liver. The limitations are the small size of the study and the unexpected heterogeneity of the patients despite the strict inclusion criteria. Also, the variable amount of ascites found at laparotomy confounded the precise estimation of bleeding and fluid shifts.

## Conclusions

A routine of generous perioperative administration of exogenous albumin during major abdominal surgery (liver transplantation), which maintained plasma albumin concentration, resulted in a cumulative albumin shift from plasma of 48 ± 33 g (*p* = 0.0017) until POD 3. This corresponds to 68% of the net albumin balance during that time period. The effects of this retention of albumin on fluid balance and postoperative recovery remain to be elucidated. On POD 3 after liver transplantation the absolute synthesis rates of albumin and fibrinogen were high, 239 ± 84 mg/kg body weight/day and 33 (5; 161) mg/kg body weight/day, respectively, which is in contrast to reports of the rates of synthesis of liver-export proteins after other major abdominal surgical procedures.

### Key Messages

- A generous exogenous albumin supply, to keep plasma albumin unaltered, resulted in a cumulative perioperative albumin shift, i.e. losses of albumin from plasma not accounted for by bleeding, losses in drains, or gains from blood products, of 37 ± 17 g at end of surgery and of 48 ± 33 g on the third day after liver transplantation.
- The cumulative perioperative albumin shift was 68% of the net amount of albumin administered and it corresponded to 38% of the intravascular albumin mass.
- Absolute synthesis rates of the liver export proteins albumin and fibrinogen were high on the third day after liver transplantation compared to reference values.

## Abbreviations

ANOVA: Analysis of variance
ASR: absolute synthesis rate
B-Hb: Blood hemoglobin
EOS: End of surgery
FSR: Fractional synthesis rate
P-alb: Plasma albumin concentration
POD: Postoperative day
TER: Transcapillary escape rate
